# Catatonia Following Chemotherapy Complicated by Acute Kidney Injury and Delirium: A Case Report

**DOI:** 10.1192/bjo.2025.10741

**Published:** 2025-06-20

**Authors:** Wei Siang Lee, Leslie Lim, Vincent Wong

**Affiliations:** Singapore General Hospital, Singapore, Singapore

## Abstract

**Aims:** Catatonia can be secondary to psychiatric or medical conditions. Previous studies have reported associations between chemotherapy and catatonia, and between taxanes (a chemotherapeutic agent) and encephalopathy. However, there have thus far been no reports linking taxanes with catatonia. We present a patient whose catatonia emerged after receiving a taxane chemotherapy agent, docetaxel, while also suffering acute kidney injury and delirium.

**Methods:** Case report.

Results:

A 75-year-old housewife was admitted to a tertiary general hospital in Singapore for delirium followed by catatonia. She had a history of a right lentiform nucleus infarct in 2017 and of schizophrenia diagnosed in 1994, and treated with haloperidol. Her schizophrenia featured auditory hallucinations, delusions, and pressured speech; but no catatonia. In May 2024, she was diagnosed with stage III left breast carcinoma and commenced on neoadjuvant chemotherapy, consisting of docetaxel, pertuzumab, trastuzumab and filgastrim. Over the next 3 weeks, she developed poor oral intake and vomiting, resulting in acute kidney injury with metabolic acidosis, and requiring admission for rehydration. Initially, she was delirious, with drowsiness and inattention. However, on the third week of admission, she developed catatonia, with features of stupor, staring, echolalia, stereotypy, verbigeration, waxy flexibility, and perseveration. On the Bush–Francis catatonia rating scale (BFCRS), she scored 23. She did not exhibit any relapse of schizophrenia. To treat her catatonia, she was prescribed PO lorazepam 0.75 mg/day to 1.5 mg/day. Serial reviews before, and 30 minutes after the administration of lorazepam, demonstrated a significant reduction in catatonic symptoms. A week later, her catatonia resolved (BFCRS score 2) and she was discharged home. During clinic follow-up, she remained haemodynamically stable with good oral intake. Her BFCRS initially increased to 10, requiring further increase of lorazepam to 3 mg/day, which led to an improvement of the BFCRS to 2.

**Conclusion:** Breast cancer chemotherapy has previously been associated with cognitive deficits. Taxane-induced neurotoxicity can present with encephalopathy, ataxia, emotional distress, or cognitive impairment. Cerebral perfusion abnormalities in the motor cortex and frontal lobe have been previously described in catatonia, and after chemotherapy for breast cancer. In contrast, pertuzumab, trastuzumab, and filgrastim lack strong association with psychological symptoms. Therefore, while the catatonia is likely multifactorial in aetiology, with dehydration, acute kidney injury, and acidosis the probable culprits, we postulate that docetaxel significantly contributed to her catatonia. Notwithstanding the pathophysiology of catatonia, the case demonstrates good response to a benzodiazepine.

